# Post‐Release Survival and Behavioral Recovery of a Rehabilitated Short‐Finned Pilot Whale ( *Globicephala macrorhynchus* ) in the South China Sea Revealed Through Satellite Tracking

**DOI:** 10.1002/ece3.73174

**Published:** 2026-03-09

**Authors:** Mingming Liu, Mingli Lin, Agathe Serres, Mingyue Ouyang, Songhai Li

**Affiliations:** ^1^ Marine Mammal and Marine Bioacoustics Laboratory, Institute of Deep‐Sea Science and Engineering Chinese Academy of Sciences Sanya China; ^2^ University of Chinese Academy of Sciences Beijing China; ^3^ The Innovation Research Center for Aquatic Mammals, Key Laboratory of Aquatic Biodiversity and Conservation of the Chinese Academy of Sciences, Institute of Hydrobiology, Chinese Academy of Sciences Wuhan China

**Keywords:** diving behavior, movement ecology, post‐release monitoring, satellite tracking, short‐finned pilot whale, South China Sea (SCS)

## Abstract

Post‐release monitoring is critical for evaluating the success of rehabilitating stranded cetaceans, yet such data are scarce for many species in the South China Sea (SCS). We satellite‐tracked a rehabilitated subadult male short‐finned pilot whale ( *Globicephala macrorhynchus* ; named “*Haitang*”), following a live stranding on Hainan Island, China. We collected tracking locations and behavioral data over a 52‐day period and conducted a dedicated 3‐day expedition to resight *Haitang* at sea in the sixth week post‐release. Results confirm *Haitang'*s survival beyond the critical 6‐week benchmark. Its daily movement distance (6.2–145.9 km) and speed (0.6–5.9 km/h) were consistent with those of healthy, free‐ranging conspecifics. Furthermore, diving behavior including time‐at‐depth budgets, maximum dive depth (621 m), diel patterns, and thermal‐depth profiles reflected natural foraging activity and environmental adaptation. Notably, high density and spatial proximity of local conspecifics during the post‐release expedition indicate favorable conditions for social integration and long‐term survival. Movement trajectories suggest that the Qiongdongnan slope represents a critical habitat for this species in the northern SCS. This study provides the first empirical evidence of successful rehabilitation and release of short‐finned pilot whale in the SCS, supporting future stranding response and conservation initiatives.

## Graphical abstract

This study presents the first empirical evidence of successful rehabilitation and post‐release monitoring of a short‐finned pilot whale in the South China Sea—a region where such data are critically lacking despite frequent stranding events. Using satellite telemetry and a dedicated resighting expedition, we documented the 52‐day movement, diving behavior, and social reintegration potential of a rehabilitated subadult male, “Haitang”. Our findings not only confirm his survival beyond the critical six‐week benchmark but also provide novel insights into the species' habitat use, diel diving patterns, and behavioral recovery in a poorly studied oceanic region.

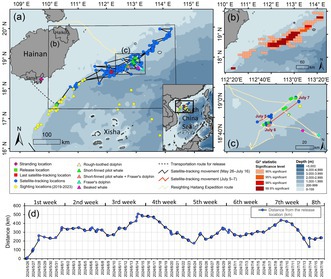

## 1 Introduction

The short‐finned pilot whale ( *Globicephala macrorhynchus* ) is a deep‐diving, highly social delphinid with a global distribution in tropical to warm temperate waters, where it shows a strong preference for continental slopes, shelf edges, and oceanic islands (García‐Aguilar et al. 2021; Jefferson et al. 2015; Olson 2018). This species is notably prone to stranding, especially in mass events, as frequently reported in many tropical‐subtropical regions like Australia (Groom and Coughran 2012), Indonesia (Mustika et al. 2022), Florida, USA (Moore et al. 2020; Wells, Fougeres, et al. 2013), and the Philippines (Aragones et al. 2024). In the South China Sea (SCS, western Pacific), the short‐finned pilot whale is relatively common, comprising approximately 10% of cetacean encounters in northern SCS surveys, and these groups typically comprised abundant individuals (mean ± SD: 45.3 ± 41.7; Lin et al. 2021; Liu, Lin, Lin, Dong, and Li 2024; Liu, Lin, Lin, Dong, Liu, et al. 2024). Furthermore, strandings of this species were regularly documented along the coasts in the SCS, particularly in the northern and western areas (Chou et al. 2024; Liu et al. 2019, 2022; Liu, Lin, Lin, Dong, and Li 2024; McGowen et al. 2021). Despite the species' prevalence in the region, no post‐release monitoring of rehabilitated individuals has been conducted in the SCS, creating a critical gap in evaluating the success of conservation interventions and the understanding of species' regional movement ecology.

Recent advances in biologging have enabled detailed behavioral and ecological studies of rehabilitated cetaceans after live strandings, transforming satellite tagging from a simple tracking tool into a critical method for validating survival and assessing long‐term outcomes (Moore et al. 2007; Sampson et al. 2012; Wells, Fauquier, et al. 2013; Zagzebski et al. 2006). Satellite‐tracking studies can not only provide definitive evidence of post‐release survival—a critical metric for conservation efforts—but also yield insights into movement ecology and behavioral patterns that are otherwise unattainable (Dunn et al. 2020; Pulis et al. 2018; Scott et al. 2001). For example, satellite tracking of rehabilitated odontocetes—spanning species such as pilot whales (*Globicephala* spp.; Moore et al. 2020; Wells, Fougeres, et al. 2013), bottlenose dolphins (*Tursiops* spp.; Balmer et al. 2010; Wells et al. 1999), and harbor porpoises ( *Phocoena phocoena* ; Schofield et al. 2008; Westgate et al. 1998)—has documented successful long‐term post‐release survival, re‐integration into natural habitats, and in some cases, remarkable long‐distance movements.

Crucially, satellite‐tracking studies of rehabilitated pilot whales can offer particularly instructive insights, revealing key adaptive behaviors and physiological benchmarks. For example, several studies have demonstrated not only survival but also the recovery of deep‐diving capacity—a vital indicator of physiological health—and the strategic use of ocean currents for efficient travel (Gales et al. 2012; Mate et al. 2005; Moore et al. 2020). These findings establish an interpretive framework in which normal movement and dive profiles serve as strong proxies for successful rehabilitation (Nawojchik et al. 2003; Wells, Fougeres, et al. 2013). However, the application of this framework remains geographically limited—most satellite‐tracking studies of rehabilitated pilot whales have occurred in the Atlantic and Oceanian basins. Consequently, little is known about the post‐release ecology of this species in the distinct oceanographic context of the western Pacific.

Our study addresses this gap directly. We deployed a satellite tag on a rehabilitated subadult male short‐finned pilot whale stranded on Hainan Island, China, applying the established monitoring framework to a novel regional context in the SCS. Our primary objectives were to: (1) evaluate the post‐release survival success of the tracked pilot whale, and (2) characterize its post‐release movement and diving behavior.

## 2 Methods

### 2.1 Stranding, Rescue, and Rehabilitation

**FIGURE 1:**
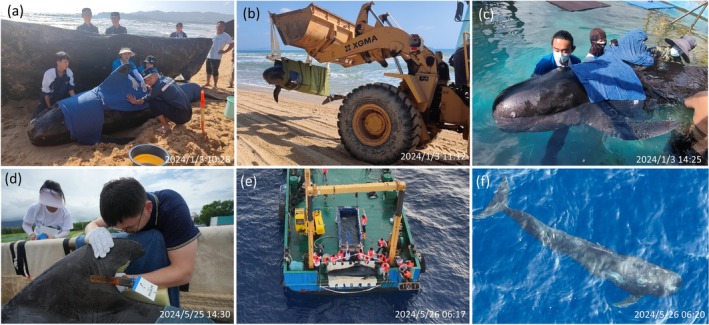
Key events of the short‐finned pilot whale ( *Globicephala macrorhynchus* ; male subadult, 3.6 m long) named “*Haitang*” recorded on Hainan Island, China in 2024: (a) stranding, (b) transportation, (c) rehabilitation, (d) satellite‐tagging and (e, f) release to wild. Photos by the Hainan Daily and the Sanya Blue Ribbon Ocean Conservation Association.

**FIGURE 2:**
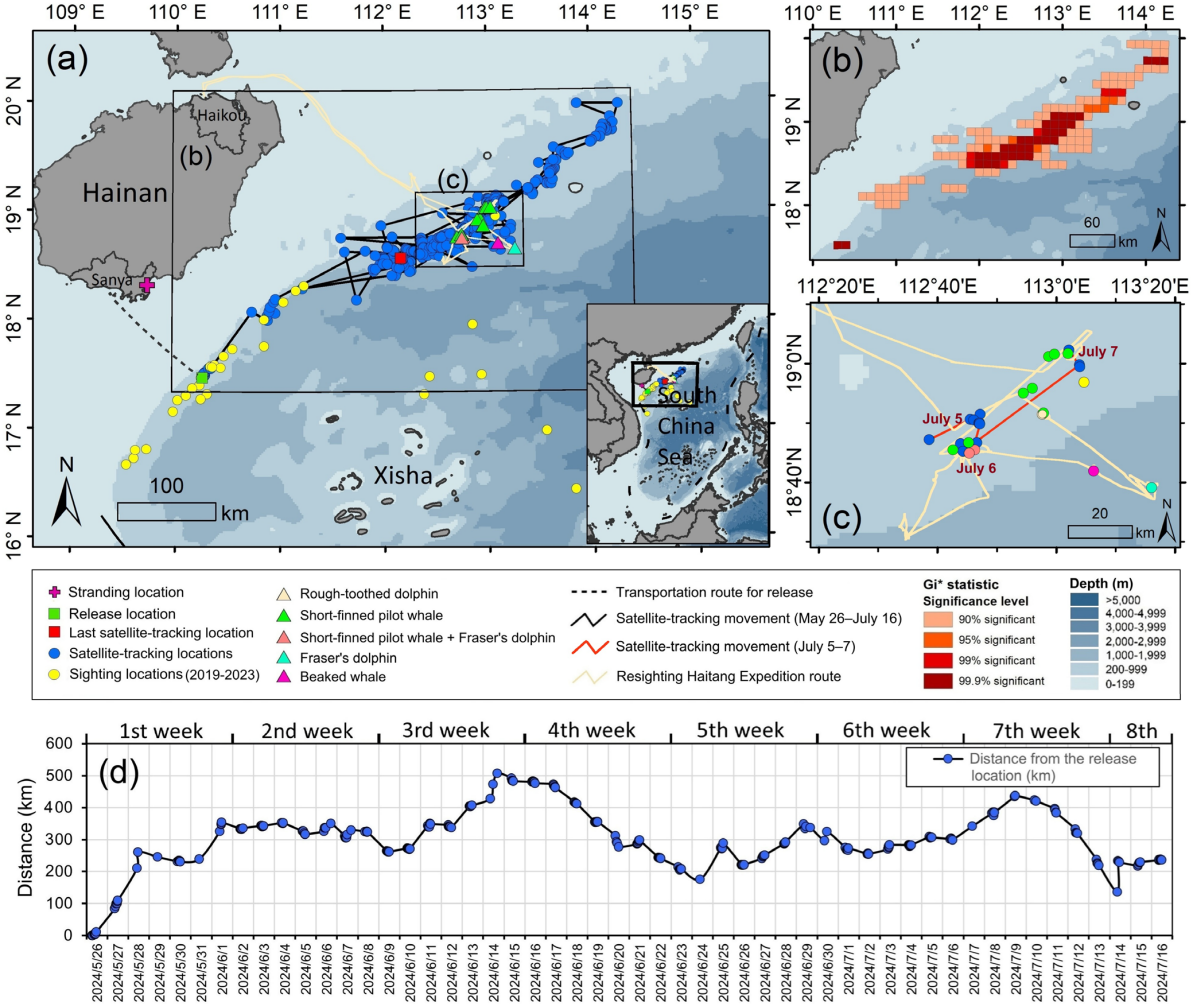
(a) *Haitang*'s satellite‐tracking locations (blue dots) and horizontal movement routes (black lines) over 52 tracking days from May 26, 2024 (release to wild) to July 16, 2024 (tag's satellite messages stopped transmitting). Yellow dots indicate encounter locations of short‐finned pilot whales previously recorded from cetacean‐dedicated surveys between 2019 and 2023 (Liu, Lin, Lin, Dong, and Li 2024; Liu, Lin, Lin, Dong, Liu, et al. 2024). (b) Hotspot areas of all satellite‐tracking locations based on Gi* hotspot analysis. (c) Survey routes (yellow lines) and encounter locations (triangle symbols) of various cetacean species recorded during the Resighting Haitang Expedition conducted between July 5–7, 2024, shown together with *Haitang*'s satellite‐tracking locations acquired during July 5–7, 2024. (d) Distance (km) measured from each satellite‐tracking location to the initial release location.

On the early morning of January 3, 2024, a live short‐finned pilot whale stranded on Haitang Bay beach in Sanya, China (Figures 1a and 2a). This individual, subsequently named “*Haitang*”, was handled by a rescue team from the Hainan Cetacean Stranding Response Network at approximately 09:30 (local time zone: UTC + 8; all times hereafter in UTC + 8 unless noted). By 14:00, the team had transported “*Haitang*” to Sanya Haichang Fantasy Town (Figure 1b,c). Two experienced veterinarians identified *Haitang* as a subadult male, measuring 3.6 m in length and weighing approximately 500 kg. Due to its difficulty maintaining natural buoyancy, *Haitang* was placed in a small circular pool (radius: ~3 m) for medical treatment and provided with supportive flotation devices (Figure 1c; Geraci and Lounsbury 2005).

On January 21, 2024, *Haitang* was transferred to a larger outdoor aquarium pond (approximately 30 m × 50 m × 3 m in length × width × depth) for rehabilitation. The period of rehabilitation lasted approximately 4 months. On May 7, 2024, a comprehensive pre‐release health assessment was conducted by an 11‐member panel composed of five veterinarians, two researchers, two NGO staff, and two government officers. *Haitang*'s health was evaluated based on physical condition (e.g., body mass/length, presence of scars, wounds, or bruising), food intake records, clinical diagnostics (e.g., gastroscopy, ultrasonography, and hematology), and behavioral monitoring (e.g., breathing interval, swimming posture, balance, and directional control) (Dierauf and Gulland 2001; Sampson et al. 2012; Zagzebski et al. 2006). Considering all these factors, the evaluation panel concluded that *Haitang* met all releasable criteria including completely healed surface injuries, normal ratio of body mass and length, sufficient food intake and good appetite (10–15 kg squid and/or herring daily), clinical parameters within typical ranges, and no abnormal behavior.

### 2.2 Satellite‐Tagging and Release

Following established protocols (Gales et al. 2012; Moore et al. 2020; Wells, Fougeres, et al. 2013), we deployed a dorsal fin‐mounted satellite tag (SPLASH10‐268D, Wildlife Computers, Redmond, WA, USA) on *Haitang*. The tag weighed 89 g in air and measured 212 × 21 × 31 mm (length × width × height). After applying topical anesthesia to the attachment site, two 8‐mm‐diameter holes were drilled through the dorsal fin using a sterilized stainless steel coring tool, positioned approximately 30 mm and 60 mm from the posterior trailing edge (Figure 1d). The tag was secured using a slow‐degrading cable tie threaded through these holes. The entire procedure was completed within 15 min on May 25, 2024, 1 day before release, with care taken to minimize *Haitang*'s struggling behavior and stress response.

*Haitang* was placed in a custom‐fitted stretcher and open container filled with recirculating seawater, hoisted aboard the research vessel “*LIYANG 358*” (500 gross tons), and transported offshore under the supervision of the Hainan Cetacean Stranding Response Network (Figure 1e). After a 16‐h transit (including 6‐h on truck and 10‐h aboard vessel), *Haitang* was released at 06:18 on May 26, 2024, at a predetermined site southeast of Sanya. The release location was selected as the nearest deep‐water habitat (water depth: around 500 m; distance: approximately 70 nm from the Sanya Bay Port and 60 nm from Hainan Island; Figure 2a) known to be used by short‐finned pilot whales in the region (Lin et al. 2021; Liu, Lin, Lin, Dong, and Li 2024; Liu, Lin, Lin, Dong, Liu, et al. 2024; Liu et al. 2026). This location minimized transportation time from land while maximizing the opportunity for *Haitang* to re‐integrate into natural habitats and locate conspecific groups. Post‐release vessel‐based monitoring continued until 07:30 to assess its initial survival and behavior in open water (Figure 1f).

### 2.3 Tag Configuration

The Splash10‐268D tag included three sensors for pressure, wet/dry status, and external temperature, along with an Argos satellite transmitter. To support long‐term monitoring, the tag's histogram sampling function for depth and ambient temperature was set to 60‐s intervals (Nawojchik et al. 2003; Wells et al. 2009). Data were aggregated into the following bins for time‐at‐depth (TAD) and time‐at‐temperature (TAT) analyses: (1) depth categories (unit: m): < 20, 20.1–50, 50.1–100, > 100; (2) temperature categories (unit: °C): < 16, 16.1–18, 18.1–20, 20.1–22, 22.1–24, 24.1–26, 26.1–28, 28.1–30, > 30. Depth bins were defined based on known dive ranges for *Globicephala* spp. (Adamczak et al. 2021; Alves et al. 2013; Shearer et al. 2022), while temperature bins reflected summertime depth‐dependent thermal gradients in the northern SCS (He et al. 2022; Huang et al. 2008).

The tag was programmed to transmit 250 times daily, balancing data acquisition and battery longevity (expected lifespan: 90–100 days; Moore et al. 2020; Wells et al. 2009). After an initial 24‐h continuous transmission period, Argos satellite positioning and data transmission were scheduled from 03:00–11:00 to 15:00–23:00 local time to coincide with satellite coverage (Dunn et al. 2020; Pulis et al. 2018; Wells et al. 2009). Satellite‐tracking locations were prioritized over depth/temperature data to ensure daily movement monitoring (Moore et al. 2020; Wells, Fougeres, et al. 2013). All other data collection features were disabled to conserve battery.

### 2.4 Post‐Release Monitoring Expedition

A dedicated at‐sea expedition (hereafter referred to as “Resighting Haitang Expedition”) was conducted from July 5 to 7, 2024, following over 5.5 weeks of stable satellite tracking, which represents a key time point for assessing long‐term survival possibility (Wells, Fauquier, et al. 2013). The primary goal was to find *Haitang* and assess its post‐release survival. Guided by recent tag locations, we implemented standard visual survey methods to search for short‐finned pilot whales and other cetaceans in the target area (Figure 2a). Observers used a reference image of *Haitang*'s tagged dorsal fin (Figure 1d) to aid at‐sea identification. During the expedition, high‐quality photos and videos were taken using Canon 7D Mark II cameras and DJI Mavic Pro drones once cetaceans were encountered (Liu, Lin, Lin, Dong, Liu, et al. 2024; Liu et al. 2026). All records were later reviewed by two experienced researchers to confirm whether *Haitang* was present.

### 2.5 Data Processing and Analyses

All original satellite messages were archived until transmissions ceased. Data were processed via Argos CLS Services (www.argos‐system.org) and the Wildlife Computers Data Portal (https://wildlifecomputers.com/). Locations from standard Argos quality classes (3, 2, 1), with estimated error radii of ≤ 250 m, 250–500 m, and 500–1500 m, respectively, were retained (Hays et al. 2001; Liu et al. 2021). Auxiliary classes (0, Z, A, B) within 5 km of a high‐quality fix were also kept based on spatial redundancy (Mate et al. 2005; Wells et al. 2009). The Douglas Argos Filter assessed location plausibility using movement speed, distance, turning angle, and location quality (Douglas et al. 2012). Locations were retained only if the derived movement speed did not exceed 20 km/h, consistent with typical horizontal swimming speeds for *Globicephala* spp. (e.g., Bloch et al. 2003; Gales et al. 2012; Moore et al. 2020; Wells, Fougeres, et al. 2013).

Based on filtered satellite‐tracking locations, hotspot areas for *Haitang* were identified using Gi* hotspot analysis in ArcGIS 10.1 (Liu et al. 2022), with a search radius of 5 km. The 52‐day satellite‐tracking period was divided into eight consecutive weeks including seven full weeks plus a three‐day eighth week. *Haitang*'s weekly movement routes were reconstructed by connecting time‐series locations within each week. Its horizontal movement distance (km) and speed (km/h) were calculated using consecutive filtered locations ≥ 30 min apart (Pulis et al. 2018; Schofield et al. 2008). Positions apart < 30 min were excluded to avoid Argos positioning inaccuracies. Straight‐line distances (km) from each satellite‐tracking location to the release, and from the first satellite‐tracking location to the last per week, were measured using “Proximity” and “Point Distance” tools in ArcGIS.

Raw depth and temperature data were decoded from Argos satellite messages using Igor Pro 9.05 (WaveMetrics, Lake Oswego, OR, USA). Stacked bar charts illustrated TAD and TAT profiles over 26 and 37 days of successful recording, respectively. Time‐series of depth and temperature measurements (10‐min intervals) were plotted over 47 and 43 tracking days. Bathymetric values at each satellite‐tracking location were extracted from the ETOPO Global Relief Model (NOAA) and overlaid with depth time‐series data to contextualize environmental variation.

To examine diel deep‐diving patterns, a 24‐h histogram (UTC + 8) was constructed showing the percentage of time spent within predefined depth layers (< 20, 20.1–50, 50.1–100, > 100; unit: m). Sunrise and sunset times for the study area (Sanya, Hainan) were obtained from an online database (https://www.timeanddate.com/), with daytime and nighttime durations averaged across all 52 tracking days (Dunn et al. 2020; Moore et al. 2020). Using OriginPro 2024 (OriginLab, Northampton, MA, USA), concurrent depth (X‐axis) and temperature (Y‐axis) records were plotted and fitted with a regression curve to explore their relationship.

## 3 Results

### 3.1 Satellite Tracking Profile

The first satellite transmission arrived 7 min post‐release, with the initial qualified location recorded approximately 350 m from the release location (Figure 2a). The tag transmitted continuously until July 16, 2024, spanning 52 tracking days. At least one qualified satellite‐tracking location was obtained daily (range: 1–12), yielding 335 qualified locations (mean: 6.4 per day and 41.8 per week). Of these locations, 120 were standard quality (3, 2, 1) and 215 were auxiliary (0, Z, A, B).

### 3.2 Movement Patterns

**FIGURE 3:**
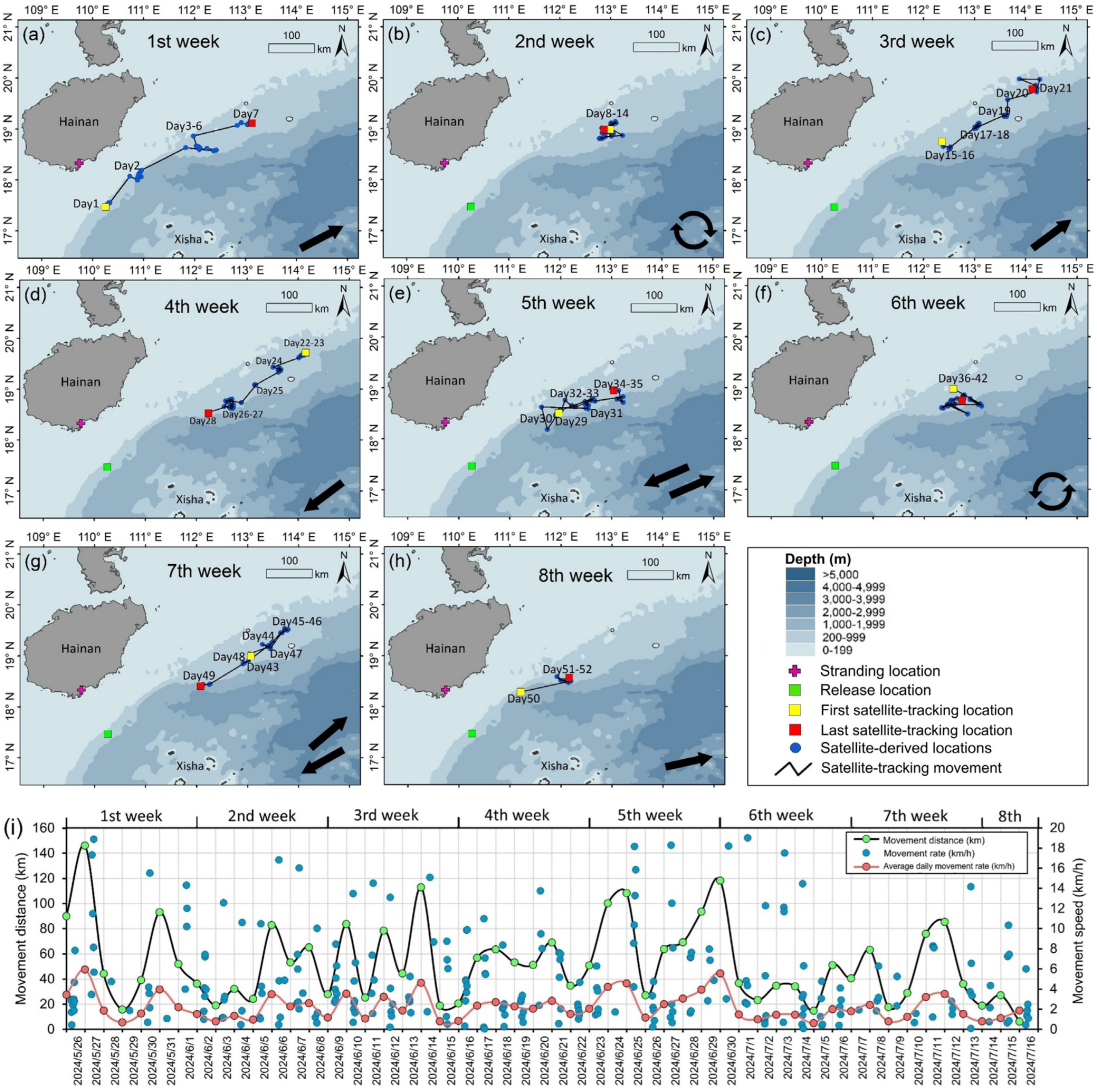
Satellite‐tracking‐ locations (blue dots) and movement routes (black lines) of *Haitang* in the (a) first, (b) second, (c) third, (d) fourth, (e) fifth, (f) sixth, (g) seventh, and (h) eighth week post‐release. Predominant movement orientation of *Haitang* marked at the right bottom corner in each panel. (i) Movement distance (km) and speed (km/h) of *Haitang* in 52 tracking days from May 26 to July 16, 2024.

Over the 52‐day satellite‐tracking period, *Haitang* traveled a minimum cumulative distance of 2757.6 km, based on all 335 filtered locations (Table 1 and Figure 2a). Gi* hotspot analysis revealed its concentrated presence along the Qiongdongnan continental slope off Hainan Island, mainly within the 200–999 m depth zone (Figure 2b). After release, *Haitang* moved northeast in the 1st week (Figure 3a), stayed in a small area within a radius of approximately 15 km in the 2nd week (Table 1 and Figure 3b), and resumed northeast movement in the 3rd week (Figure 3c). It reversed to the southwest in the 4th week (Figure 3d), showed oscillatory movement in the 5th week (Figure 3e), and restricted movement to a radius of approximately 30 km in the 6th week, overlapping spatially with the 2nd week's range (Table 1 and Figure 3f). It resumed variable directional movement in the 7th and 8th weeks (Figure 3g,h).

**TABLE 1:** Summary of movement characteristics calculated using satellite‐tracking data acquired from “*Haitang*”, a live‐stranded and rehabilitated short‐finned pilot whale ( *Globicephala macrorhynchus* ) during its 52 post‐release tracking days in the South China Sea. Ocean depth (m) measured at each satellite‐tracking location from a bathymetric layer of the ETOPO Global Relief Model.

Week post release	Period	No. of days	No. of qualified satellite‐tracking locations	Horizontal movement distance (km; Figure 3a–h)	Horizontal movement speed (km/h; Figure 3i)	Distance from first location to last (km; Figure 3a–h)	Distance from last location to release (km; Figure 3a–h)	Ocean depth (m, mean ± SD; Figure 4c)
1st	May 26, 2024–June 1, 2024	7	37	480.0	2.8	353.8	354.0	513.7 ± 221.3
2nd	June 2, 2024–June 8, 2024	7	49	312.4	1.9	11.9	323.5	484.3 ± 119.4
3rd	June 9, 2024–June 15, 2024	7	49	391.1	2.3	219.4	482.6	550.2 ± 108.2
4th	June 16, 2024–June 22, 2024	7	54	349.4	2.1	241.3	240.6	570.9 ± 131.2
5th	June 23, 2024–June 29, 2024	7	49	512.6	3.1	124.5	336.9	647.1 ± 287.5
6th	June 30, 2024–July 6, 2024	7	46	312.8	1.9	29.6	298.8	627.6 ± 264.7
7th	July 7, 2024–July 13, 2024	7	31	347.3	2.1	124.2	219.3	634.0 ± 271.2
8th	July 14, 2024–July 16, 2024	3	20	52.0	1.0	104.7	235.7	621.9 ± 229.7
Total	May 26, 2024–July 16, 2024	52	335	2757.6	2.2	104.7	235.7	576.7 ± 216.3

The mean distance from the 335 filtered satellite‐tracking locations to the release location was 297.0 ± 99.5 km (mean ± SD; Figure 2a,d). The farthest point (506.9 km from release) was recorded on June 14, 2024 (Day 21; Figures 2d and 3c). The last satellite‐tracking location on July 16, 2024 (Day 52) was 235.7 km from release (Figures 2d and 3h). Daily movement distances ranged from 6.2 to 145.9 km (mean ± SD: 53.0 ± 31.3 km; Figure 3i). The highest weekly cumulative movement distance (512.6 km) was observed in the 5th week (Table 1 and Figure 3e). Daily movement distances exceeded 100 km on only 5 days (Day 2, 20, 30, 31, and 36; Figure 3i). The overall movement speed was 2.2 km/h (i.e., 52.8 km/day). Acceptable movement speed (≤ 20 km/h) varied from 0.1 to 18.9 km/h, with daily averages between 0.6 and 5.9 km/h (Figure 3i).

### 3.3 Resighting Haitang Expedition

The Resighting Haitang Expedition (July 5–7, 2024) included three survey days with 13 cetacean encounters: eight short‐finned pilot whale groups, two mixed‐species groups of pilot whales and Fraser's dolphins ( *Lagenodelphis hosei* ), one Fraser's dolphin group, one rough‐toothed dolphin ( *Steno bredanensis* ) group, and one unidentified beaked whale (Ziphiidae) (Table 2 and Figure 2c). Short‐finned pilot whale group sizes ranged from 10–20 to approximately 100 individuals, with encounter location depths averaging 558.5 ± 83.2 m (mean ± SD, *n* = 10; Table 2). The 10 pilot whale encounters occurred 0.5–18.8 km from *Haitang*'s nearest satellite‐tracking locations (Table 2 and Figure 2c), with distance of only 0.2–6.2 km on July 6–7, 2024 (Table 2). Despite this close proximity of these groups to *Haitang*'s transmitted locations and the collection of 12,491 high‐quality photographs and 164.4 min of drone videos, we did not confirm *Haitang*'s presence among any individuals. This was likely due to the large group sizes and the challenge posed by satellite transmission‐reception delays in pinpointing the individual's real‐time location.

**TABLE 2:** Cetacean groups encountered during the Resighting Haitang Expedition carried out during July 5–7, 2024.

Date	Encounter time (UTC + 8)	Species^a^ (group size)	Distance to nearest satellite‐tracking location (km)	Water depth (m)	Number of high‐quality photographs	Duration of drone videos (mins)
2024/7/5	13:28–14:49	GM (10–30)	17.7	522	3385	33.4
2024/7/5	14:51–15:27	GM (10–20)	14.3	454	131	4.9
2024/7/5	16:01–16:15	SB (20–40)	18.1	708	384	10.9
2024/7/5	16:29–16:48	GM (~100)	18.8	684	555	5.0
2024/7/6	05:30–06:05	LH (> 100)	53.8	1380	716	9.2
2024/7/6	08:26–08:29	ZP (1)	35.3	1258	0	0
2024/7/6	10:48–11:31	GM (~50) + LH (100–200)	2.0	663	2404	21.2
2024/7/6	11:33–12:51	GM (~20)	2.7	604	1097	3.9
2024/7/6	13:00–13:37	GM (40–50)	2.3	589	854	25.4
2024/7/6	15:19–18:18	GM (~50) + LH (~100)	1.8	640	690	17.1
2024/7/7	12:35–12:50	GM (~20)	0.2	463	428	8.2
2024/7/7	12:56–13:06	GM (10–20)	6.2	467	383	3.8
2024/7/7	13:26–15:55	GM (> 30)	4.3	502	1464	21.4

^a^ Species abbreviations: GM (short‐finned pilot whale, *Globicephala macrorhynchus* ), SB (rough‐toothed dolphin, *Steno bredanensis* ), LH (Fraser's dolphin, *Lagenodelphis hosei* ), and ZP (unidentified beaked whale, Ziphiidae).

### 3.4 Depth and Temperature Variability

**FIGURE 4:**
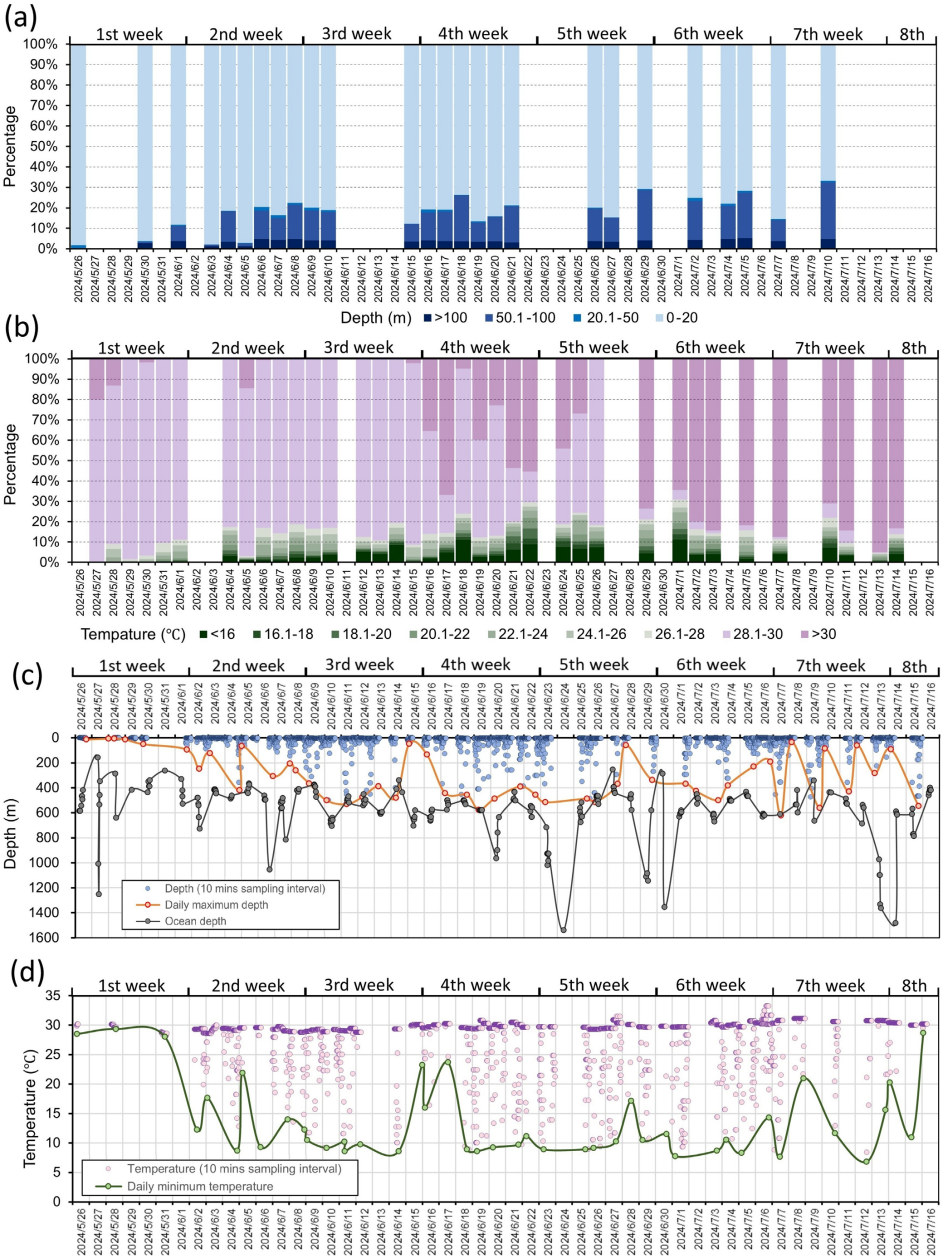
Satellite‐tracking profiles of *Haitang* in 52 post‐release days from May 26 to July 16, 2024: (a) TAD (time at depth) and (b) TAT (time at temperature) data summaries, and time‐series (c) depth and (d) temperature measurements. TAD and TAT data summaries indicated proportion of duration *Haitang* stayed at four depth ranges (unit: M): 0–20, 20.1–50, 50.1–100, and > 100, and at nine temperature ranges (unit: °C): < 16, 16.1–18, 18.1–20, 20.1–22, 22.1–24, 24.1–26, 26.1–28, 28.1–30, and > 30, respectively. Time‐series data were collected for depth (blue dots) and temperature (purple dots) measurements with 10‐min sampling intervals. Daily maximum depth shown by orange circles and daily minimum temperature by green circles. Ocean depth (gray dots) measured at each satellite‐tracking location from a bathymetric layer of the ETOPO Global Relief Model and weekly summarized in Table 1.

TAD and TAT analyses indicated *Haitang* primarily occupied the upper 20 m of the water column (Figure 4a) and temperatures ≥ 28.1°C (Figure 4b). Time spent at depths > 20 m and temperatures < 28°C was limited (daily proportion < 10%) initially, but increased and stabilized at 10%–30% in subsequent weeks (Figure 4a,b). The TAD daily proportion in depths greater than 20 m rose from 1.8% (May 26) to 33.2% (July 10; Figure 4a), while the TAT proportion in temperatures lower than 28°C increased from 0% (May 27) to a peak of 30.8% (July 2; Figure 4b).

Weekly maximum dive depths were 95.3 m (1st week), 418.8 m (2nd week), 531.0 m (3rd week), 575.8 m (4th week), 515.8 m (5th week), 500.8 m (6th week), 621.0 m (7th week), and 545.5 m (8th week), maintaining around 500 m since the 2nd week (Figure 4c). Ocean depths at the 335 filtered satellite‐tracking locations ranged from 157 to 1539 m (mean ± SD: 576.7 ± 216.3 m), showing a weak increase with time (Table 1; Figure 4c). Minimum weekly temperatures were 28.0°C (1st week), 8.7°C (2nd week), 8.6°C (3rd week), 8.6°C (4th week), 8.9°C (5th week), 7.8°C (6th week), 6.8°C (7th week), and 11.0°C (8th week) (Figure 4d).

**FIGURE 5:**
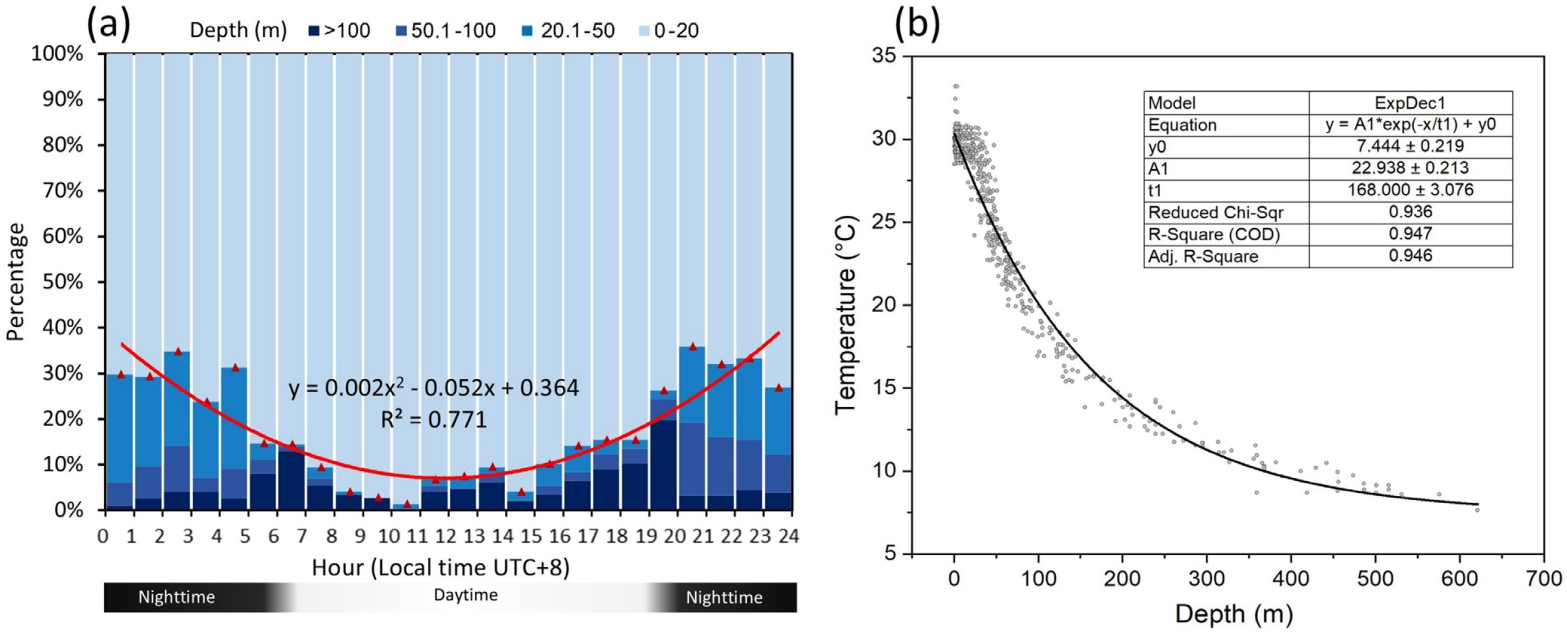
(a) Diel pattern of TAD (time at depth) data shown by the percentage of time spent within predefined depth layers (< 20, 20.1–50, 50.1–100, > 100; unit: M) using a 24‐h histogram (UTC + 8) across 52 tracking days. (b) Regression of concurrent depth (X‐axis) and temperature (Y‐axis) records fitted with an exponential curve.

Diel depth patterns showed *Haitang* spent 1.4%–15.4% of daytime (05:00–19:00) at depths > 20 m, increasing to approximately 30% at night (19:00–05:00; Figure 5a). The TAD proportion for depths > 20 m was fitted with a quadratic function ($y=0.002x^{2}-0.052x+0.364;R^{2}=0.771$; Figure 5a). A strong exponential relationship ($y=A_{1}*\exp\left(\frac{-x}{t_{1}}\right)+y_{0};R^{2}\left(\mathrm{COD}\right)=0.947$) was observed between concurrent depth and temperature records (*n* = 2411; Figure 5b).

## 4 Discussion

### 4.1 Survival Success

First, *Haitang*'s 52‐day post‐release satellite‐tracking period supports a successful survival outcome. Based on an evaluation of 69 cases across 10 odontocete species, Wells, Fauquier, et al. (2013) proposed a benchmark of at least 6 weeks post‐release survival for rehabilitated cetaceans, as individuals surviving beyond this period demonstrate a high probability of long‐term survival. With a tracking duration of 52 days (i.e., 7.5 weeks), *Haitang* meets this criterion, indicating a positive rehabilitation achievement. Tracking duration serves as one indicator of post‐release success, though it is influenced by tag performance, attachment integrity, and the individual's health status (Andrews et al. 2019; Moore et al. 2020; Wells, Fougeres, et al. 2013). The 52‐day period was substantially shorter than the tag's expected battery life (i.e., 90–100 days). A status report received 8 days before transmissions ceased indicated sufficient battery capacity, with only approximately 14,000 of an expected 25,000 transmissions completed. Therefore, battery exhaustion is unlikely to have caused transmission termination, which was more likely due to preprogrammed tag detachment.

Second, the recovery of species‐typical movement and diving behaviors further substantiates *Haitang*'s long‐term survival prospects. During the first week, *Haitang*'s movement was directionally consistent toward the northeast with very scarce dives exceeding 100 m, a pattern potentially indicative of broad‐scale searching behavior for conspecifics or familiar habitats (Schofield et al. 2008; Moore et al. 2020). After the first week, movement became more variable, time spent at depths > 20 m increased to 10%–30%, and daily maximum dive depths regularly reached > 200 m. Together, these behavioral shifts, including a diel pattern of increased deep‐diving at night, align closely with known species‐specific ecology (Aguilar Soto et al. 2008; Alves et al. 2013; Quick et al. 2017), supporting the conclusion that *Haitang* successfully adapted to post‐release life in its natural habitat.

Third, findings from the Resighting Haitang Expedition provided supplementary evidence confirming *Haitang*'s survival into the sixth week post‐release. During the expedition, 13 cetacean groups were encountered, 10 of which consisted of short‐finned pilot whales totaling several hundred individuals. This high local abundance suggests favorable conditions for long‐term survival, given the social and foraging benefits of group integration (Hill et al. 2019; Mahaffy et al. 2015; Wells, Fougeres, et al. 2013). Over the three‐day expedition, *Haitang*'s movements were confined to an area with a radius of approximately 30 km, with the nearest conspecific groups located only a few kilometers from *Haitang*'s satellite‐tracking positions. This spatial proximity further suggests that *Haitang* was well‐positioned to integrate into a free‐ranging conspecific group, although direct photographic confirmation was not obtained.

### 4.2 Movement, Distribution, and Habitat

The combination of *Haitang*'s satellite‐tracking routes and identified hotspot areas with data from the Resighting Haitang Expedition extends the known distribution range of short‐finned pilot whales in the waters nearby Hainan Island, which have not been fully investigated during boat‐based surveys (Lin et al. 2021; Liu, Lin, Lin, Dong, and Li 2024; Liu, Lin, Lin, Dong, Liu, et al. 2024). Although released within a previously recognized hotspot based on 23 ship‐based encounters (Figure 2a), *Haitang* did not stay near the release location. Instead, it rapidly departed the area and exhibited a combination of directional travel, temporary residency, and highly variable movement. The overall average movement speed (i.e., 2.2 km/h) and average daily speed (0.6–5.9 km/h) align with published values for the species: 0.2–14.5 km/h (Bloch et al. 2003), 2–7 km/h (Wells, Fougeres, et al. 2013), 3.4 km/h (0.03–9.98; García‐Aguilar et al. 2021), 3.4 km/h (0.03–9.98; García‐Aguilar et al. 2021), 4.4 km/h (Moore et al. 2020), and 6 km/h (Gales et al. 2012). Although conservative, these movement estimates collectively indicate *Haitang*'s healthy locomotor performance after its release.

*Haitang*'s habitat use was consistent with the species' known preference for continental slope environments (Kendall‐Bar et al. 2016; Moore et al. 2020; Thorne et al. 2017). Mean water depths derived from 335 qualified satellite‐tracking locations (576.7 ± 216.3 m) and those recorded during the Resighting Haitang Expedition (558.5 ± 83.2 m; *n* = 10) closely matched previously reported bathymetric ranges in the region (e.g., 462.8 ± 140.4 m to 1025.5 ± 655.4 m; Liu, Lin, Lin, Dong, and Li 2024). These results indicate that the area frequented by *Haitang* represents a recurrent and significant habitat for the species. Combined with recent survey data (Liu, Lin, Lin, Dong, and Li 2024; Liu, Lin, Lin, Dong, Liu, et al. 2024), this study identifies the Qiongdongnan slope as a critical habitat for short‐finned pilot whales in the northern SCS.

### 4.3 Deep‐Diving Behavior

TAD analysis revealed that during the first week post‐release, *Haitang* spent > 90% of its time in the upper 20 m of the water column and performed few deep dives. A progressive recovery of deep‐diving behavior was observed in subsequent weeks, reflected by increased time at depths greater than 20 m, greater maximum dive depths, and lower minimum temperatures. From the second week onward, daily time budgets spent at depths > 20 m stabilized between 10% and 30%, with a slight increasing trend over time. These patterns are consistent with rehabilitated conspecifics, which spend roughly 80%–90% of their time near the sea surface (Mate et al. 2005; Wells, Fauquier, et al. 2013), and align with natural behavior in free‐ranging individuals: approximately 75%–85% of time in the upper 20‐m layers (Alves et al. 2013; Heide‐Jørgensen et al. 2002; Quick et al. 2017). Notably, *Haitang*'s gradually increased maximum recorded dive depth (418.8–621.0 m) since the second post‐release week falls within the known range recorded from healthy, free‐ranging conspecifics including 540–1018 m (Aguilar Soto et al. 2008), 130–988 m (Alves et al. 2013), 600–828 m (Heide‐Jørgensen et al. 2002), 621.4 ± 225.8 m (max: 864 m; Gough et al. 2025), and 444 ± 85 (max: 617 m; Aoki et al. 2017), confirming the restoration of its deep‐diving capacity.

Time‐budget analyses also revealed a clear diel pattern: *Haitang* spent approximately twice as much time at depths > 20 m during nighttime compared to daytime. This finding is consistent with previously reported nocturnal diving behavior in pilot whales (Moore et al. 2020; Nawojchik et al. 2003; Wells, Fougeres, et al. 2013). This pattern aligns with the hypothesis that deep‐diving odontocetes are more active at night, likely in association with foraging on vertically migrating prey (Alves et al. 2013; Quick et al. 2017; Shearer et al. 2022). Specifically, pilot whales may perform shallower, non‐feeding dives during the day when the deep scattering layer is at greater depths, and deeper dives at night to access prey as the layer ascends (Baird et al. 2002; Gough et al. 2025; Heide‐Jørgensen et al. 2002).

By using *Haitang* as a mobile sampling platform, vertical movement data revealed temperature variations along the bathymetric gradient (i.e., 0–600 m) in the Qiongdongnan slope area during summer, showing a strong exponential relationship. This result is highly consistent with in situ measurements and physical oceanographic models from the northern SCS (He et al. 2022; Huang et al. 2008), though thermal profiles may differ in other regions due to latitudinal and environmental gradients (Adamczak et al. 2021).

### 4.4 Conclusion and Perspectives

In summary, we conclude that *Haitang*'s release and subsequent survival were successful, based on three lines of evidence: (1) a 52‐day satellite‐tracking duration exceeding the six‐week survival benchmark, (2) high conspecific abundance near *Haitang*'s satellite‐tracking locations during the Resighting Haitang Expedition in the sixth week post‐release, and (3) the recovery of species‐typical behavioral patterns in movement, deep‐diving time budgets, diel dive rhythms, and maximum dive depth.

Despite this success, behavioral and ecological insights derived from a single rehabilitated individual remain limited. Future efforts in the SCS should prioritize satellite‐tagging opportunities for live‐stranded and rehabilitated short‐finned pilot whales, given the abundance of this species and the frequency of stranding events in the region. Tracking more individuals will enhance the evaluation of rehabilitation and release outcomes while providing valuable opportunities to investigate species‐specific movement and behavioral ecology that are difficult to assess through traditional visual and acoustic surveys alone.

## Author Contributions

**Mingming Liu:** conceptualization, data curation, formal analysis, funding acquisition, investigation, methodology, software, validation, visualization, writing – original draft, writing – review and editing. **Mingli Lin:** investigation, methodology, writing – review and editing. **Agathe Serres:** data curation, investigation, writing – review and editing. **Mingyue Ouyang:** data curation, investigation, writing – review and editing. **Songhai Li:** conceptualization, funding acquisition, investigation, methodology, project administration, resources, supervision, validation, writing – review and editing.

## Funding

This work was supported by the National Natural Science Foundation of China (42225604 and 42494883), the Science and Technology Talent Innovation Project of Hainan (KJRC2023B03), and the Hainan Provincial Natural Science Foundation of China (424RC535).

## Ethics Statement

This research was performed under an Ethical Statement (IDSSE‐SYLL‐MMMBL‐01) approved by the Institute of Deep‐sea Science and Engineering, Chinese Academy of Sciences, and an Official Permit (No. 2020–1726) from the Hainan Provincial Department of Agriculture and Rural Affairs, People's Republic of China.

## Conflicts of Interest

The authors declare no conflicts of interest.

## Data Availability

The data that support the findings of this study are available via the Dryad data publishing platform (DOI: 10.5061/dryad.8cz8w9h5x).

## References

[ece373174-bib-0001] Adamczak, S. K. , W. A. McLellan , A. J. Read , C. L. Wolfe , and L. H. Thorne . 2021. “The Impact of Temperature at Depth on Estimates of Thermal Habitat for Short‐Finned Pilot Whales.” Marine Mammal Science 37, no. 1: 193–206. 10.1111/mms.12737.

[ece373174-bib-0002] Aguilar Soto, N. , M. P. Johnson , P. T. Madsen , et al. 2008. “Cheetahs of the Deep Sea: Deep Foraging Sprints in Short‐Finned Pilot Whales Off Tenerife (Canary Islands).” Journal of Animal Ecology 77, no. 5: 936–947. 10.1111/j.1365-2656.2008.01393.x. 18444999

[ece373174-bib-0003] Alves, F. , A. Dinis , C. Ribeiro , et al. 2013. “Daytime Dive Characteristics From Six Short‐Finned Pilot Whales *Globicephala macrorhynchus* Off Madeira Island.” Arquipélago. Life and Marine Sciences 31: 1–8.

[ece373174-bib-0004] Andrews, R. D. , R. W. Baird , J. Calambokidis , et al. 2019. “Best Practice Guidelines for Cetacean Tagging.” Journal of Cetacean Research and Management 20, no. 1: 27–66. 10.47536/jcrm.v20i1.237.

[ece373174-bib-0005] Aoki, K. , K. Sato , S. Isojunno , T. Narazaki , and P. J. Miller . 2017. “High Diving Metabolic Rate Indicated by High‐Speed Transit to Depth in Negatively Buoyant Long‐Finned Pilot Whales.” Journal of Experimental Biology 220, no. 20: 3802–3811. 10.1242/jeb.158287. 29046419

[ece373174-bib-0006] Aragones, L. V. , A. N. L. Morado , M. C. M. Obusan , et al. 2024. “Spatiotemporal Variation of Stranded Marine Mammals in The Philippines From 2005 to 2022: Latest Stranding Hotspots and Species Stranding Status.” Aquatic Mammals 50, no. 4: 302–322. 10.1578/AM.50.4.2024.302.

[ece373174-bib-0007] Baird, R. W. , J. F. Borsani , M. B. Hanson , and P. L. Tyack . 2002. “Diving and Night‐Time Behavior of Long‐Finned Pilot Whales in the Ligurian Sea.” Marine Ecology Progress Series 237: 301–305. 10.3354/meps.

[ece373174-bib-0008] Balmer, B. C. , L. H. Schwacke , and R. S. Wells . 2010. “Linking Dive Behavior to Satellite‐Linked Tag Condition for a Bottlenose Dolphin (*Tursiops truncatus*) Along Florida's Northern Gulf of Mexico Coast.” Aquatic Mammals 36, no. 1: 1–8. 10.1578/AM.36.1.2010.1.

[ece373174-bib-0009] Bloch, D. , M. P. Heide‐Jørgensen , E. Stefansson , et al. 2003. “Short‐Term Movements of Long‐Finned Pilot Whales *Globicephala melas* Around the Faroe Islands.” Wildlife Biology 9, no. 1: 47–58. 10.2981/wlb.2003.007.

[ece373174-bib-0010] Chou, L. S. , C. J. Yao , M. C. Wang , W. L. Chi , Y. Ho , and W. C. Yang . 2024. “Cetacean Stranding Response Program and Spatial‐Temporal Analysis in Taiwan, 1994–2018.” Animals 14, no. 12: 1823. 10.3390/ani14121823. 38929442 PMC11200669

[ece373174-bib-0011] Dierauf, L. , and F. M. Gulland , eds. 2001. CRC Handbook of Marine Mammal Medicine: Health, Disease, and Rehabilitation. CRC Press.

[ece373174-bib-0012] Douglas, D. C. , R. Weinzierl , S. C. Davidson , R. Kays , M. Wikelski , and G. Bohrer . 2012. “Moderating Argos Location Errors in Animal Tracking Data.” Methods in Ecology and Evolution 3, no. 6: 999–1007. 10.1111/j.2041-210X.2012.00245.x.

[ece373174-bib-0013] Dunn, C. , D. Claridge , D. Herzing , et al. 2020. “Satellite‐Linked Telemetry Study of a Rehabilitated and Released Atlantic Spotted Dolphin in The Bahamas Provides Insights Into Broader Ranging Patterns and Conservation Needs.” Aquatic Mammals 46, no. 6: 633–639. 10.1578/AM.46.6.2020.633.

[ece373174-bib-0014] Gales, R. , R. Alderman , S. Thalmann , and K. Carlyon . 2012. “Satellite Tracking of Long‐Finned Pilot Whales ( *Globicephala melas* ) Following Stranding and Release in Tasmania, Australia.” Wildlife Research 39, no. 6: 520–531. 10.1071/WR12023.

[ece373174-bib-0015] García‐Aguilar, M. C. , M. A. Pardo , A. Fajardo‐Yamamoto , M. R. Ramírez‐León , and O. Sosa‐Nishizaki . 2021. “First Insights on the Horizontal Movements of Short‐Finned Pilot Whales in the Gulf of Mexico.” Marine Ecology 42, no. 3: e12656. 10.1111/maec.12656.

[ece373174-bib-0016] Geraci, J. R. , and V. J. Lounsbury . 2005. Marine Mammals Ashore: A Field Guide for Strandings. National Aquarium in Baltimore.

[ece373174-bib-0017] Gough, W. T. , B. C. Madrigal , A. Hollers , et al. 2025. “Daily Energetic Expenditure and Energy Consumption of Short‐Finned Pilot Whales.” Journal of Experimental Biology 228, no. 21: jeb249821. 10.1242/jeb.249821. 41232179

[ece373174-bib-0018] Groom, C. J. , and D. K. Coughran . 2012. “Three Decades of Cetacean Strandings in Western Australia: 1981 to 2010.” Journal of the Royal Society of Western Australia 95, no. 1: 63–77.

[ece373174-bib-0019] Hays, G. C. , S. Åkesson , B. J. Godley , P. Luschi , and P. Santidrian . 2001. “The Implications of Location Accuracy for the Interpretation of Satellite‐Tracking Data.” Animal Behaviour 61, no. 5: 1035–1040. 10.1006/anbe.2001.1685.

[ece373174-bib-0020] He, Z. , W. Hu , L. Li , T. Pähtz , and J. Li . 2022. “Thermohaline Dynamics in the Northern Continental Slope of the South China Sea: A Case Study in the Qiongdongnan Slope.” Journal of Marine Science and Engineering 10, no. 9: 1221. 10.3390/jmse10091221.

[ece373174-bib-0021] Heide‐Jørgensen, M. P. , D. Bloch , E. Stefansson , B. Mikkelsen , L. Helen Ofstad , and R. Dietz . 2002. “Diving Behaviour of Long‐Finned Pilot Whales *Globicephala melas* Around the Faroe Islands.” Wildlife Biology 8, no. 4: 307–313.

[ece373174-bib-0022] Hill, M. C. , A. R. Bendlin , A. M. van Cise , et al. 2019. “Short‐Finned Pilot Whales (Globicephala Macrorhynchus) of the Mariana Archipelago: Individual Affiliations, Movements, and Spatial Use.” Marine Mammal Science 35, no. 3: 797–824. 10.1111/mms.12567.

[ece373174-bib-0023] Huang, B. , W. Lan , Z. Cao , et al. 2008. “Spatial and Temporal Distribution of Nanoflagellates in the Northern South China Sea.” Hydrobiologia 605, no. 1: 143–157. 10.1007/s10750-008-9330-3.

[ece373174-bib-0024] Jefferson, T. A. , M. A. Webber , and R. L. Pitman . 2015. Marine Mammals of the World: A Comprehensive Guide to Their Identification, 193–196. Academic Press.

[ece373174-bib-0025] Kendall‐Bar, J. M. , D. W. Weller , H. Fearnbach , et al. 2016. “Movement and Occurrence Patterns of Short‐Finned Pilot Whales ( *Globicephala macrorhynchus* ) in the Eastern North Pacific.” Aquatic Mammals 42, no. 3: 300–305. 10.1578/AM.42.3.2016.300.

[ece373174-bib-0026] Lin, M. , M. Liu , F. Caruso , et al. 2021. “A Pioneering Survey of Deep‐Diving and Off‐Shore Cetaceans in the Northern South China Sea.” Integrative Zoology 16, no. 4: 440–450. 10.1111/1749-4877.12508. 33259136

[ece373174-bib-0027] Liu, M. , M. Lin , and S. Li . 2022. “Species Diversity and Spatiotemporal Patterns Based on Cetacean Stranding Records in China, 1950–2018.” Science of the Total Environment 822: 153651. 10.1016/j.scitotenv.2022.153651. 35124055

[ece373174-bib-0028] Liu, M. , M. Lin , P. Zhang , T. Xue , and S. Li . 2019. “An Overview of Cetacean Stranding Around Hainan Island in the South China Sea, 1978–2016: Implications for Research, Conservation and Management.” Marine Policy 101: 147–153. 10.1016/j.marpol.2018.04.029.

[ece373174-bib-0031] Liu, M. , W. Lin , M. Lin , et al. 2021. “The First Attempt of Satellite Tracking on Occurrence and Migration of Bryde's Whale ( *Balaenoptera edeni* ) in the Beibu Gulf.” Journal of Marine Science and Engineering 9, no. 8: 796. 10.3390/jmse9080796.

[ece373174-bib-0029] Liu, M. , W. Lin , M. Lin , L. Dong , and S. Li . 2024. “Short‐Finned Pilot Whales in the South China Sea: Insights Into Regional Distribution, Movement Pattern, and Habitat Characteristics.” Marine Mammal Science 40, no. 1: 89–107. 10.1111/mms.13052.

[ece373174-bib-0030] Liu, M. , W. Lin , M. Lin , et al. 2024. “Species Diversity and Critical Habitats of Offshore and Deep‐Diving Cetaceans in the South China Sea.” Biological Conservation 299: 110808. 10.1016/j.biocon.2024.110808.

[ece373174-bib-0032] Liu, M. , M. Ouyang , W. Lin , et al. 2026. “Diverse Mixed‐Species Cetacean Groups in the South China Sea: First Documentation From A Potential Important Marine Mammal Area.” Marine Mammal Science 42, no. 1: e70093. 10.1111/mms.70093.

[ece373174-bib-0033] Mahaffy, S. D. , R. W. Baird , D. J. McSweeney , D. L. Webster , and G. S. Schorr . 2015. “High Site Fidelity, Strong Associations, and Long‐Term Bonds: Short‐Finned Pilot Whales Off the Island of Hawai'i.” Marine Mammal Science 31, no. 4: 1427–1451. 10.1111/mms.12234.

[ece373174-bib-0034] Mate, B. R. , B. A. Lagerquist , M. Winsor , J. Geraci , and J. H. Prescott . 2005. “Movements and Dive Habits of a Satellite‐Monitored Long‐Finned Pilot Whale (*Globicephala melas*) in the Northwest Atlantic.” Marine Mammal Science 21, no. 1: 136–144. 10.1111/j.1748-7692.2005.tb01213.x.

[ece373174-bib-0035] McGowen, M. R. , L. Vu , C. W. Potter , et al. 2021. “Whale Temples Are Unique Repositories for Understanding Marine Mammal Diversity in Central Vietnam.” Raffles Bulletin of Zoology 69: 481–496. 10.26107/RBZ-2021-0066.

[ece373174-bib-0036] Moore, M. , G. Early , K. Touhey , S. Barco , F. Gulland , and R. Wells . 2007. “Rehabilitation and Release of Marine Mammals in the United States: Risks and Benefits.” Marine Mammal Science 23, no. 4: 731–750. 10.1111/j.1748-7692.2007.00146.x.

[ece373174-bib-0037] Moore, R. B. T. , D. C. Douglas , H. H. Nollens , L. Croft , and R. S. Wells . 2020. “Post‐Release Monitoring of a Stranded and Rehabilitated Short‐Finned Pilot Whale (*Globicephala macrorhynchus*) Reveals Current‐Assisted Travel.” Aquatic Mammals 46, no. 2: 200–214. 10.1578/AM.46.2.2020.200.

[ece373174-bib-0038] Mustika, P. L. K. , K. K. High , M. I. H. Putra , et al. 2022. “When and Where Did They Strand? The Spatio‐Temporal Hotspot Patterns of Cetacean Stranding Events in Indonesia.” Oceans 3, no. 4: 509–526. 10.3390/oceans3040034.

[ece373174-bib-0039] Nawojchik, R. , D. J. S. Aubin , and A. Johnson . 2003. “Movements and Dive Behavior of Two Stranded, Rehabilitated Long‐Finned Pilot Whales ( *Globicephala melas* ) in the Northwest Atlantic.” Marine Mammal Science 19, no. 1: 232–239. 10.1111/j.1748-7692.2003.tb01105.x.

[ece373174-bib-0040] Olson, P. A. 2018. “Pilot Whales: *Globicephala melas* and *G. macrorhynchus* .” In Encyclopedia of Marine Mammals, 3rd ed., 701–705. Academic Press. 10.1016/B978-0-12-804327-1.00194-1.

[ece373174-bib-0041] Pulis, E. E. , R. S. Wells , G. S. Schorr , D. C. Douglas , M. M. Samuelson , and M. Solangi . 2018. “Movements and Dive Patterns of Pygmy Killer Whales (*Feresa attenuata*) Released in the Gulf of Mexico Following Rehabilitation.” Aquatic Mammals 44, no. 5: 555–567. 10.1578/AM.44.5.2018.555.

[ece373174-bib-0042] Quick, N. J. , S. Isojunno , D. Sadykova , M. Bowers , D. P. Nowacek , and A. J. Read . 2017. “Hidden Markov Models Reveal Complexity in the Diving Behaviour of Short‐Finned Pilot Whales.” Scientific Reports 7, no. 1: 45765. 10.1038/srep45765. 28361954 PMC5374633

[ece373174-bib-0043] Sampson, K. , C. Merigo , K. Lagueux , et al. 2012. “Clinical Assessment and Post‐Release Monitoring of 11 Mass Stranded Dolphins on Cape Cod, Massachusetts.” Marine Mammal Science 28, no. 4: E404–E425. 10.1111/j.1748-7692.2011.00547.x.

[ece373174-bib-0044] Schofield, T. D. , G. Early , F. W. Wenzel , et al. 2008. “Rehabilitation and Homing Behavior of a Satellite‐Tracked Harbor Porpoise (*Phocoena phocoena*).” Aquatic Mammals 34, no. 1: 1–8. 10.1578/AM.34.1.2008.1.

[ece373174-bib-0045] Scott, M. D. , A. A. Hohn , A. J. Westgate , J. R. Nicolas , B. R. Whitaker , and W. B. Campbell . 2001. “A Note on the Release and Tracking of A Rehabilitated Pygmy Sperm Whale (*Kogia breviceps*).” Journal of Cetacean Research and Management 3, no. 1: 87–94.

[ece373174-bib-0046] Shearer, J. M. , F. H. Jensen , N. J. Quick , et al. 2022. “Short‐Finned Pilot Whales Exhibit Behavioral Plasticity in Foraging Strategies Mediated by Their Environment.” Marine Ecology Progress Series 695: 1–14. 10.3354/meps14132.

[ece373174-bib-0047] Thorne, L. H. , H. J. Foley , R. W. Baird , D. L. Webster , Z. T. Swaim , and A. J. Read . 2017. “Movement and Foraging Behavior of Short‐Finned Pilot Whales in the Mid‐Atlantic Bight: Importance of Bathymetric Features and Implications for Management.” Marine Ecology Progress Series 584: 245–257. 10.3354/meps12371.

[ece373174-bib-0049] Wells, R. S. , D. A. Fauquier , F. M. Gulland , F. I. Townsend , and R. A. DiGiovanni . 2013. “Evaluating Postintervention Survival of Free‐Ranging Odontocete Cetaceans.” Marine Mammal Science 29, no. 4: E463–E483. 10.1111/mms.12007.

[ece373174-bib-0050] Wells, R. S. , E. M. Fougeres , A. G. Cooper , et al. 2013. “Movements and Dive Patterns of Short‐Finned Pilot Whales (*Globicephala macrorhynchus*) Released From a Mass Stranding in the Florida Keys.” Aquatic Mammals 39, no. 1: 61–72. 10.1578/AM.39.1.2013.61.

[ece373174-bib-0051] Wells, R. S. , C. A. Manire , L. Byrd , et al. 2009. “Movements and Dive Patterns of a Rehabilitated Risso's Dolphin, *Grampus griseus* , in the Gulf of Mexico and Atlantic Ocean.” Marine Mammal Science 25, no. 2: 420–429. 10.1111/j.1748-7692.2008.00251.x.

[ece373174-bib-0052] Wells, R. S. , H. L. Rhinehart , P. Cunningham , et al. 1999. “Long Distance Offshore Movements of Bottlenose Dolphins.” Marine Mammal Science 15, no. 4: 1098–1114. 10.1111/j.1748-7692.1999.tb00879.x.

[ece373174-bib-0053] Westgate, A. J. , A. J. Read , T. M. Cox , T. D. Schofield , B. R. Whitaker , and K. E. Anderson . 1998. “Monitoring A Rehabilitated Harbor Porpoise Using Satellite Telemetry.” Marine Mammal Science 14, no. 3: 599–604. 10.1111/j.1748-7692.1998.tb00746.x.

[ece373174-bib-0054] Zagzebski, K. A. , F. M. Gulland , M. Haulena , and M. E. Lander . 2006. “Twenty‐Five Years of Rehabilitation of Odontocetes Stranded in Central and Northern California, 1977 to 2002.” Aquatic Mammals 32, no. 3: 334–345. 10.1578/AM.32.3.2006.334.

